# Supplementary material for: Prevalence of timely complementary feeding initiation and associated factors among mothers having children aged 6–24 months in rural north-central Ethiopia: Community based cross-sectional study
Source: PLoS One. 2022 May 18;17(5):e0267008. doi: 10.1371/journal.pone.0267008 (PMC9116650; doi:10.1371/journal.pone.0267008)
Supplement: S1 File — (DOCX) [file pone.0267008.s001.docx]

## English Version Questionnaire Information Sheet

Questionnaire identification number________________

Note: Circle the number from the given options and write if any other idea or answer is given.

Part-1: Demographic and Socio-economic characteristics

| No | Questions | Answer | Skip |
| --- | --- | --- | --- |
| 101 | Age of the mother | _________ in years |  |
| 102 | Marital status | 1. Single 2. Married 3. Divorced 4. Widowed 5. Separated |  |
| 103 | Religion | 1. Orthodox 2. Muslim 3. Protestant 4. Other (specify) _____________ |  |
| 104 | Educational status of the mother | 1. Unable to read and write 2. Able to read and write 3. Primary education (grade1-8) 4. secondary education (9-12) 5. TVT/diploma and above |  |
| 105 | Educational status of spouse | 1. Unable to read and write 2. Able to read and write 3. Primary education(grade 1-8) 4. secondary education(grade 9-12) 5. TVT/diploma and above |  |
| 106 | Occupation of the mother | 1. Housewife 2. Keep livestock 3. Daily laborer 4. Merchant 5. Governmental employee 6. Other(specify)______________ |  |
| 107 | Occupation of the husband | 1. Farmer 2. Daily laborer 3. Merchant 4. Governmental employee 5. Other (specify)_____________ |  |
| 108 | Access of Radio | 1. Yes 2. No |  |
| 109 | Read magazines and books(at least once per month) | 1. Yes 2. No |  |
| 110 | Monthly income of the household | ________Ethiopian birr |  |
| 111 | Family size | ________ in number |  |
| 112 | Child’s sex | 1. Male  2. Female |  |

**Part-II Maternal care and birth history**

| 201 | Number of live births | _______ in years |  |
| --- | --- | --- | --- |
| 202 | Birth intervals | __________years |  |
| 203 | Did you visit health facility for ANC during your pregnancy for this child? | 1. Yes 2. No |  |
| 204 | How many times did you visit? | 1. One times 2. Two times 3. Three times 4. Four times |  |
| 205 | During (any of) ANC visit(s), were you told about the time to complementary feeding? | 1. Yes 2. No |  |
| 206 | Where did you deliver the current child/children/Place of current delivery? | 1. Home 2. Government health facility 3. Private health facility 4. Other (specify)__________ |  |
| 207 | Did you visit health facility for PNC for this child? | 1. Yes 2. No |  |
| 208 | During (any of) PNC visit(s), were you told about the time to complementary feeding? | 1. Yes 2. No |  |
| 209 | How many children delivered in the last birth? | 1. Only one 2. More than one |  |

**Part-III Maternal source of information and knowledge of mothers about timing of introducing complementary foods to their children**

| 301 | Did you seek an advice about infant feeding practice? | 1. Yes 2. No |  |
| --- | --- | --- | --- |
| 302 | If your response is yes on question number 301 where did you seek? | 1. Colleagues/relatives 2. Family 3. Health extension worker 4. Government health facility 5. Private health facility 6. Media (radio, TV, magazines) 7. Other (specify)__________ |  |
| 303 | Did you give the child pre-lactation food/fluid? | 1. Yes 2. No | If no skip to 306 |
| 304 | If your response is yes on question number 303 What did you give for him (her)? | 1. Butter 2. Salt solution 3. Cow’s milk 4. water 5. Other (specify)_________ |  |
| 305 | What was the reason for introducing prelacteal feed? | 1. Breast milk insufficiency 2. Culture of the community 3. Maternal illness 4. Caesarean delivery 5. painful breast 6. Others (specify)__________ |  |
| 306 | Have you started offering additional food for this last child? | 1. Yes 2. No |  |
| 307 | When did you start? (First introduce complementary food to the child). | 1. __________in months 2. Do not know_______ |  |
| 308 | If a mother starts complementary feeding to her infant before six months, the possible reason(s) is/are | 1. Maternal illness 2. painful breast 3. Mother feel that her breast milk is not enough 4. Mother feel that the child is large enough to take additional food 5. Since the child is small 6. Due to twins births 7. Need for another new born 8. Since mother had additional work outside the home 9. Do not know exactly when to start 10. Other (specify)___________ | Skip to 309 if not applicable |
| 309 | If a mother starts complementary feeding to her infant after six months latter, the possible reason(s) is/are | 1. Mother feel that only her breast milk is enough up to one year of age 2. Mother feel that the child not large enough to take additional food 3. The husband advice not start additional food before one year of age 4. Community culture is not allowed for offering additional food before one year old 5. child illness 6. Lack of appropriate food 7. Did not know exactly when to start 8. Other (specify)____________ |  |
