# Supplementary material for: Prevalence of timely complementary feeding initiation and associated factors among mothers having children aged 6–24 months in rural north-central Ethiopia: Community based cross-sectional study
Source: PLoS One. 2022 May 18;17(5):e0267008. doi: 10.1371/journal.pone.0267008 (PMC9116650; doi:10.1371/journal.pone.0267008)
Supplement: Attachment — Submitted filename: PONE-D-21-34084.docx [file pone.0267008.s002.docx]

PLOS ONE

Prevalence of Timely Complementary Feeding Initiation and Associated Factors

among Mothers Having Children Aged 6-24 Months in Rural North-central Ethiopia:

Community based Cros-sectional Study

--Manuscript Draft--

| Manuscript Number: | PONE-D-21-34084 |
| --- | --- |
| Article Type: | Research Article |
| Full Title: | Prevalence of Timely Complementary Feeding Initiation and Associated Factors among Mothers Having Children Aged 6-24 Months in Rural North-central Ethiopia:  Community based Cross-sectional Study |
| Short Title: | Timely Complementary Feeding Initiation and Associated Factors among Mothers Having Children Aged 6-24 Months |
| Corresponding Author: | Moges WUBNEH ABATE, MSc  Debre Tabor University  Debre Tabor, ETHIOPIA |
| Keywords: | Complementary feeding, Timely inititations, Rural, Ethiopia. |
| Abstract: | Background : The timely initiation of complementary foods during infancy is necessary for growth and development. In the past years, despite efforts put to increase timely initiation of complementary feeding among lactating mothers in Ethiopia by different stakeholders, the goal is not attain as expected.  Objective: The main aim of this study was to assess the prevalence of timely initiation of complementary feeding among mothers having children aged 6-24 months in Farta district, rural Ethiopia.  Methods : A community-based cross-sectional study was employed from December  2020 to February 2021 among 570 mothers by using multi-stage sampling techniques. Data were collected using a structured interviewer-administered questionnaire and entered into Epi Data 4.6 then transferred to Statistical Package for Social Science version 25 for analysis. Bivariate and multivariable logistic regression analysis with a 95% confidence interval carried out to determine the association between explanatory and the outcome variables. A P-value of < 0.05 was considered statistically significant.  Results : The prevalence of timely initiation of complementary feeding among mothers was 52.1%. Institutional delivery [(AOR=2.10, 95% CI: (1.31-3.32)], Keeping livestock [(AOR=2.21, 95% CI: (1.35, 3.65)], Postnatal follow up [(AOR=0.60, 95% CI: (0.36,  0.77)], merchants [(AOR=4.58; 95% CI: 1.99, 10.55)], and daily laborer [(AOR=2.88, 95% CI: (1.50-5.51)] were statistically associated with timely initiation of complementary feeding.  Conclusion: This finding revealed that the prevalence of timely initiation of  complementary feeding is still low. Factors affecting timely initiations of complementary feeding were home delivery, unable to attend postnatal care follow-up, being housewife and farmers. |
| Order of Authors: | Moges WUBNEH ABATE, MSc |
|  | ADANE BIRHANU NIGAT, MSc |
|  | AGIMASIE TIGABU DEMELASH, MSc |
|  | TIGABU DESIE EMIRU, MSc |
|  | NIGUSIE SELOMON TIBEBU, MSc |
|  | CHALIE MAREW TIRUNEH, MSc |
|  | AMSALU BELETE MEKONEN, MSc |
|  | TILAHUN KEGNE ABEBE, MPH |
|  |  |

|  | MOGES YINGES YITAYEW, MPH |
| --- | --- |
| Additional Information: |  |
| Question | Response |
| Financial Disclosure  Enter a financial disclosure statement that describes the sources of funding for the work included in this submission. Review the [submission guidelines](http://journals.plos.org/plosone/s/submission-guidelines#loc-financial-disclosure-statement) for detailed requirements. View published research articles from [PLOS ONE](http://journals.plos.org/plosone/) for specific examples.  This statement is required for submission and will appear in the published article if the submission is accepted. Please make sure it is accurate. | The author(s) received no specific funding for this work |
| Unfunded studies Enter: The author(s) received no specific funding for this work. Funded studies Enter a statement with the following details: - Initials of the authors who received each award - Grant numbers awarded to each author - The full name of each funder - URL of each funder website - Did the sponsors or funders play any role in the study design, data collection and analysis, decision to publish, or preparation of the manuscript? - NO - Include this sentence at the end of your statement: The funders had no role in study design, data collection and analysis, decision to publish, or preparation of the manuscript. - YES - Specify the role(s) played. |  |
| * typeset |  |
| Competing Interests  Use the instructions below to enter a competing interest statement for this submission. On behalf of all authors, disclose any [competing interests](http://journals.plos.org/plosone/s/competing-interests) that could be perceived to bias this work—acknowledging all financial support and any other relevant financial or nonfinancial competing interests. | The authors have declared that no competing interests exist |

| This statement is required for submission and will appear in the published article if the submission is accepted. Please make sure it is accurate and that any funding sources listed in your Funding Information later in the submission form are also declared in your Financial Disclosure statement.  View published research articles from [PLOS ONE](http://journals.plos.org/plosone/) for specific examples. |  |
| --- | --- |
| NO authors have competing interests Enter: The authors have declared that no competing interests exist. Authors with competing interests Enter competing interest details beginning with this statement: I have read the journal's policy and the authors of this manuscript have the following competing interests: [insert competing interests here] |  |
| * typeset |  |
| Ethics Statement  Enter an ethics statement for this submission. This statement is required if the study involved:   - Human participants - Human specimens or tissue - Vertebrate animals or cephalopods - Vertebrate embryos or tissues - Field research   Write "N/A" if the submission does not require an ethics statement.  General guidance is provided below. Consult the [submission guidelines](http://journals.plos.org/plosone/s/submission-guidelines#loc-guidelines-for-specific-study-types) for detailed instructions. Make sure that all | Ethical approval was obtained from ethical review board of Bahir Dar University with a protocol number 00310/2020. A formal permission letter was communicated at each level. The participants were well informed about the purpose of the study and the right to refuse participation and information was collected after oral and written consent from each respondent. |

information entered here is included in the

Methods section of the manuscript.

Format for specific study types

Human Subject Research (involving human

participants and/or tissue)

Give the name of the institutional review

board or ethics committee that approved the

study

•

Include the approval number and/or a

statement indicating approval of this

research

•

Indicate the form of consent obtained

(

written/oral) or the reason that consent was

not obtained (e.g. the data were analyzed

anonymously)

•

Animal Research (involving vertebrate

animals, embryos or tissues)

Provide the name of the Institutional Animal

Care and Use Committee (IACUC) or other

relevant ethics board that reviewed the

study protocol, and indicate whether they

approved this research or granted a formal

waiver of ethical approval

•

Include an approval number if one was

obtained

•

If the study involved

non-human primates

,

add

additional details

about animal welfare

and steps taken to ameliorate suffering

•

If anesthesia, euthanasia, or any kind of

animal sacrifice is part of the study, include

briefly which substances and/or methods

were applied

•

Field Research

Include the following details if this study

involves the collection of plant, animal, or

other materials from a natural setting:

Field permit number

•

Name of the institution or relevant body that

granted permission

•

Data Availability

Authors are required to make all data

underlying the findings described fully

available, without restriction, and from the

time of publication. PLOS allows rare

exceptions to address legal and ethical

Yes - all data are fully available without restriction

| concerns. See the [PLOS Data Policy](http://journals.plos.org/plosone/s/data-availability) and [FAQ](http://journals.plos.org/plosone/s/data-availability#loc-faqs-for-data-policy) for detailed information.  A Data Availability Statement describing where the data can be found is required at submission. Your answers to this question constitute the Data Availability Statement and will be published in the article, if accepted. |  |
| --- | --- |
| Important: Stating ‘data available on request from the author’ is not sufficient. If your data are only available upon request, select ‘No’ for the first question and explain your exceptional situation in the text box. |  |
| Do the authors confirm that all data underlying the findings described in their manuscript are fully available without restriction? |  |
| Describe where the data may be found in full sentences. If you are copying our sample text, replace any instances of XXX with the appropriate details.   - If the data are held or will be held in a public repository, include URLs, accession numbers or DOIs. If this information will only be available after acceptance, indicate this by ticking the box below. For example: All XXX files   are available from the XXX database (accession number(s) XXX, XXX.).   - If the data are all contained within the manuscript and/or Supporting Information files, enter the following: All relevant data are within the manuscript and its Supporting Information files. - If neither of these applies but you are able to provide details of access elsewhere, with or without limitations, please do so. For example:   Data cannot be shared publicly because of [XXX]. Data are available from the XXX Institutional Data Access / Ethics Committee (contact via XXX) for researchers who meet the criteria for access to confidential data. | The data are available from the corresponding author and will be accessed as needed. |

| The data underlying the results presented in the study are available from (include the name of the third party and contact information or URL).  • This text is appropriate if the data are owned by a third party and authors do not have permission to share the data.  * typeset |  |
| --- | --- |
| Additional data availability information: | Tick here if the URLs/accession numbers/DOIs will be available only after acceptance of the manuscript for publication so that we can ensure their inclusion before publication. |

Manuscript	[Click here to access/download;Manuscript;manuscript.docx](https://www.editorialmanager.com/pone/download.aspx?id=30040072&guid=388b2d78-01f1-41ef-901a-c54dbbe7c1a3&scheme=1)

Prevalence of Timely Complementary Feeding Initiation and Associated Factors among Mothers Having Children Aged 6-24 Months in Rural North-central

Ethiopia: Community based Cros-sectional Study

Moges Wubneh Abate^1^, Adane Birhanu Nigat^1^, Agimasie Tigabu Demelash^1^, Tigabu Desie

Emiru^2^, Nigusie Selomon Tibebu^2^, Chalie Marew Tiruneh^2^, Amsalu Belete Mekonen^3^, Tilahun

Kegne Abebe^4^, Moges Yinges Yitayew^5^,

# Authors’ description

^1^Departments of Adult Health Nursing, College of health sciences, Debre Tabor University,

Ethiopia

^2^Departments of pediatrics and child health Nursing, College of health sciences, Debre Tabor- University, Ethiopia

^3^Departments of mental health, School of medicine, Debre Tabor University, Ethiopia

^4^Departments of public health, Debre Tabor comprehensive specialised hospital, Ethiopia

^5^Departments of public health, Ethiopian Red Cross-Society, Ethiopia

**Corresponding author: Moges Wubneh Abate,** Email: wmoges7@gmail.com

# Abstract

**Background**: The timely initiation of complementary foods during infancy is necessary for growth and developments. In the past years, despite efforts put to increase timely initiation of complementary feeding among lactating mothers in Ethiopia by different stakeholders, the goal is not attain as expected.

**Objective:** The main aim of this study was to assess the prevalence of timely initiation of complementary feeding among mothers having children aged 6-24 months in Farta district, rural Ethiopia.

**Methods**: A community-based cross-sectional study was employed from December 2020 to February 2021 among 570 mothers by using multi-stage sampling techniques. Data were collected using a structured interviewer-administered questionnaire and entered into Epi Data 4.6 then transferred to Statistical Package for Social Science version 25 for analysis. Bivariate and multivariable logistic regression analysis with a 95% confidence interval carried out to determine the association between explanatory and the outcome variables. A P-value of < 0.05 was considered statistically significant.

**Results**: The prevalence of timely initiation of complementary feeding among mothers was 52.1%. Institutional delivery [(AOR=2.10, 95% CI: (1.31-3.32)], Keeping livestock [(AOR=2.21, 95% CI: (1.35, 3.65)], Postnatal follow up [(AOR=0.60, 95% CI: (0.36, 0.77)], merchants [(AOR=4.58; 95% CI: 1.99, 10.55)], and daily laborer [(AOR=2.88, 95% CI: (1.50-

5.51)] were statistically associated with timely initiation of complementary feeding.

**Conclusion:** This finding revevaled that the prevalence of timely initiation of complementary feeding is still low. Factors affecting timely initiations of complementary feeding were home delivery, unable to attend postnatal care follow-up, being housewife and farmers.

**Keywords:** Complementary feeding, Timely inititations, Rural, Ethiopia.

## Introduction Background

Complementary foods are defined as any solid or liquid foods with a nutritional value other than breast milk, offered to breastfed infants (1).

Timely initiations of additional foods at early childhood is essential for growth and developmental reasons, and to enable the transition from milk feeding to family foods (2). Exclusive breastfeeding for six months charge by complementary feeding such as offering solid foods besides breast milk is an optimum infant feeding practice, recommended since 2002 by the World Health Organization (3). The initiations of solid food to the diet of an infant, known as complementary feeding (4). The world health organization (WHO) recommended exclusive breastfeeding for the first six months of life and mothers should initiate soft, semi-solid, or solid food for their infants at the age of six months (5). The first two years of life involve rapid physical, cognitive and social development that requires optimal nutrition (6).

It is well known that the period from birth to two years of life is a “critical window” for the optimal growth, and development (7).

In the world, near to eleven million infants and young children death annually occur due to the absence of timely initiations of complementary feeding (8). In 2017, the world prevalence of timely initiation of complementary feeding was 64.5% (9).

In developing countries, over two-hundred million children under five years unable to reach their capacity in cognitive development because of not initated timely complementary feeding (10). The immediate consequences of poor nutrition during early childhood age include morbidity, mortality and delayed mental development (11).

Ethiopia is still faced, undernourished children. This is mainly due to the absence of timely complementary feeding. In the past years, despite efforts that have been put to increase timely initiation of complementary feeding practice in Ethiopia, by different stakeholders, the goal is not attain as expected. The main purpose of this study was to assess timely initiation of complementary feeding among mothers having children aged 6-24 months in Farta district, Rural Ethiopia.

## Materials and Methods Study Area and period

The study was conducted in Farta district, which is one of the 13 districts found in South Gondar zone, Amhara, Ethiopia. It has 36 rural kebeles. There are 8 health centres and 36 health posts providing health service for the district population. The total population of Farta district is 250,731. Of these total populations, 17,664 children were under two years of age.

## Study Design and source population

A community-based cross-sectional study was conducted among mothers who have Children 6– 24 months of age in Farta district, South Gondar zone, Northcentral Ethiopia.

**Eligibility criteria Inclusion criteria**

All mothers with children aged from six months to two years in Farta district were included.

## Exclusion criteria

Mothers who will seriously ill and unable to provide information during data collection period were excluded.

**Variables**

**Dependent Variable:** Timely initiation of complementary feeding

## Independent Variables

**Socio-demographic and economic characteristics**: age of mothers, occupation, educational level, marital status, gender of the children, religion, media access, and income.

**Maternal reproductive history and health service utilization**: ANC follow up, number of visits, PNC follow up, place of delivery, number of live birth, birth interval, number of children delivered at a time.

## Operational definition

**Timely initiation of complementary feeding**: It is the initiation of additional supplementary food for a young child at sixth months of age along with continuing breast feeding (12).

## Sample size, sampling techniques and procedure

By taking the prevalence of timely initiation of complementary feeding 34% (13). Epi info version 7 .2.2.6 was used to calculate the sample by considered confidence interval 95%, power 80% with design effect 1.5. Based on this the sample size was 518. By adding a 10% nonresponse rate the final sample size was 570. A multi-stage sampling technique was used to select the study participants. First, 8 out of 36 Farta districts were selected randomly using lottery method. There were 2198 households in the selected kebeles having under-two-years-old children. The calculated sample size was proportionally allocated to the selected kebeles based on the number of households. In the second stage, a systematic random sampling technique was used to selected mothers at each selected kebeles. The sampling frame was obtained from the health extension workers at each selected kebeles. Finally, proportionally allocated study participants were selected by using systematic random sampling every 4^th^ interval while the first household was selected by lottery method.

## Data collection tool

Structured interviewer-administered questionnaires were used to collect the timely initiation of complementary feeding and its associated factors. The questionnaire was adapted from key indicators recommended by WHO and IYCF strategy of Ethiopia (12, 14). The questionnaire composed of socio-demographic and economic characteristics, health care, and complementary feeding practice-related variables. It was initially prepared in English and then translated from English to Amharic and re-translated back to English by a language expert to maintain its consistency. Pre-testing was done on 5% of mothers at Ebinat district to check the reliability of the tool. Data were collected by eight health extension workers and two masters’ nurses’ supervisor for monitoring the overall data collection process. One day of training was given to data collectors and supervisors.

### Data quality assurance, processing and analysis

Adequate training was provided for eight data collectors and two supervisors. The codes were given to the questionnaires. Data collectors and supervisors checked the filled questionnaire for completeness every day. Problems encountered during the study period were discussed in the study team and solved. Data were first checked for completeness and then each completed questionnaire assigned a unique code. Subsequently, the data were entered using Epi Data 4.6. The generated data was exported to a statistical package for social sciences (SPSS) version 25. The data was cleaned by visualizing, calculating frequencies, and sorting. The analysis was done with descriptive statistics by using frequency and percentage while bivariate and multivariable regression analysis between dependent and independent variables were performed using binary logistic regression. Hosmer and Lemeshow test was checked for model goodness of fit. During the bivariate analysis 0.2 p-values were taken into multivariable regression. A 95% confidence interval (CI) and a P-value of less than 0.05 was taken as a significant association. Results were presented in text and tables.

# Results

## Socio-demographic and economic characteristics

From a total of 570 samples, 536 mothers who have 6-24 months old children have participated in the study with a response rate of 94%. Regarding educational status respondents, diploma and above were 47 (8.8%), 86 (16%) had completed primary education (**Table 1**).

Table 1: Socio-demographic characteristics of mothers in Farta district (n=536), Ethiopia, 2021.

| Variables | Category | Frequency (N) | Percent (%) |
| --- | --- | --- | --- |
| Age of mother in years | 15-19  20-25  26-29  >=30 | 36  157 90  253 | 6.7  29.3  16.8  47.2 |
| Educational status of mother | Unable to read and write able to read and write  Primary  Secondary  Diploma and above | 115  266  86  22  47 | 21.5  49.6  16.0  4.1  8.8 |
| Educational status of spouse | Unable to read and write able to read and write  Primary  Secondary education  Diploma and above | 131  217  144  21  23 | 24.4  40.5  26.9  3.9  4.3 |
| Occupation of mother | Housewife  Keep livestock  Daily labourer  Merchant  Governmental employee | 275  121  83  26  31 | 51.3  22.6  15.5  4.9  5.8 |
| Occupation of spouse | Farmer  Governmental employee  Merchant daily labourer | 405  20  53  58 | 75.6  3.7  9.9  10.8 |
| Read magazines and books (at least one month) | Yes No | 41  495 | 7.6  92.4 |
| Monthly income of the household | ≤500 ETB  501-1000 ETB  1001-1500 ETB  1501-2000 ETB  ≥2001 ETB | 192  170  4  87  83 | 35.8  31.7 0.7  16.2  15.5 |
| Family size in number | 2-3 family  4-6 family  > 6 family | 404  103  29 | 75.4  19.2  5.4 |

**Abbrevation:** ETB= Ethiopian Birr

## Maternal reproductive history and health service utilization

Among the respondents, 315 (58.8%) of the mothers were primiparous. More than half of the mothers 313 (58.4 %) had a history of ANC follow up (**Table 2**).

**Table 2: Maternal reproductive history and health service utilization of mothers in Farta district, Ethiopia, 2020.**

| Variables | Categories | Frequency (N=536) | Percent (%) |
| --- | --- | --- | --- |
| Number of live births | ≤ 2  3-4  ≥ 5 | 315  185  36 | 58.8  34.5  6.7 |
| Birth interval | ≤ 2 years  ≥ 3 years | 273  263 | 50.9  49.1 |
| ANC follow up | Yes  No | 313  223 | 58.4  41.6 |
| Number of ANC follow up | 1-2 times  ≥ 3 times | 159  154 | 29.7  28.7 |
| During ANC visit told time to CF | Yes  No | 265  123 | 68  32 |
| Place of current delivery | Home  Institutional | 119  417 | 22.2  77.8 |
| PNC follow up | Yes  No | 262  274 | 48.9  51.1 |
| During PNC visit told time to CF | Yes  No | 200 62 | 37.3  11.6 |
| Number of children delivered. | 1  ≥ 1 | 432  104 | 80.6  19.4 |

**Abbrevations: ANC=Antenatal care, CF=Complementary Feeding, PNC= Postnatal Care**

## Timely initiation of complementary feeding

From the total participants 278 (51.9%) of mothers were initiated complementary feeding at six months.

## Factors associated with timely initiation of complementary feeding

In the multivariable logistic regression analysis, occupations of the mothers, occupation of the spouse, Place of current delivery and PNC follow up were significantly associated with timely initiation of complementary feeding. Those mothers who had not postnatal care follow-up were 40% less likely to initiate timely complementary feeding. Mothers had merchant husbands were 4.58 times more likely to initiate timely complementary feeding than mothers whose husband were farmers [(AOR=4.58; 95% CI :( 1.99, 10.55)] (**Table 3**).

Table 3: Factors associated with timely initiation of complementary feeding among mothers who had 6–24 months children in farta district.

| Variables | Categories | Timely initiation complementary feeding | | COR (95% CI) | AOR (95%CI) |
| --- | --- | --- | --- | --- | --- |
|  |  | No | Yes |  |  |
| participant occupation | Housewife  Keep livestock  Daily labourer  Merchant  Employee | 154  34  45 8  17 | 121  87  38  18  14 | 1  3.26 (2.05-5.17)**  1.08 (0.66-1.76)  2.86 (1.20-6.81) *  1.05 (0.50-2.21) | 1  2.21(1.33-3.68)**  0.79 (0.46-1.36)  1.02 (0.32-3.28)  0.97 (0.42-2.22) |
| Occupation of the spouse | Farmer  Employee  Merchant  Daily labourer | 221  13  8  16 | 184  7  45  42 | 1  0.65 (0.253-1.56)  6.76(3.11-14.70)**  3.15 (1.72-5.79)** | 1  0.54 (0.20-1.45)  4.58(1.99-10.55)**  2.88 (1.50-5.51) ** |
| Place of delivery | Home  Institutional | 75  183 | 44  234 | 1  2.18 (1.43-3.31)** | 1  2.10(1.31-3.32)** |
| PNC follow up | Yes No | 104  154 | 158  120 | 1  0.51 (0.36-0.72)** | 1  0.60(0.41-0.89)** |

**Note:** 1=Reference group, ***P≤ 0.05=** statistically significant**, **P≤ 0.01=** highly statistically significant

Hosmer-Lemeshow goodness of fit test= 0.57

**Abbrevations**: AOR= Adjusted odd ratio, CI= Confidence interval, COR= Crude odd ratio

# Discussions

The main reason to conduct this study was to assess timely initiation of complementary feeding in Farta district, rural Ethiopia. Mothers who had children 6-24 months were the study participant.

The result of this study revealed that the prevalence of timely initiation of complementary feeding was 51.9% [(95% CI; (48%, 56%)]. The timely initiation of complementary feeding practice in this study was higher than findings from Iran (44.8%) (15), Kamba district (40.4%) (16). This difference is because of in Iran the study participants were those mothers had children up to two and a half year of life which may prone to recall bias compared to our study. Besides this in Iran low birth weight, as well as multiple birth children, were excluded in the study this could also underestimate the finding. The possible reason for variation with Kamba Woreda may be due to difference in the study period since the study in Kamba was done before six year ago and nowadays the health extension workers play a great role in the dissemination of information about child feeding practice for the community. The finding was lower than the study conducted in Bangladesh (71%) (17), Halaba Kulito (57.8%) (18), Dessie Referral Hospital (65.1%) (19) and Addis Ababa (83%) (20), Hiwot Fana specialized hospital (60.5%) (21), Mekelle (62.8%) (22), Lalibela (63%) (23). This incongruent finding is due to timely initiation of complementary feeding finding in Bangladesh included those mothers who initiated feeding in 6-8 months compared with our study exactly at 6 months. Also differences in Addis Ababa, Hiwot Fana hospital, Mekelle and Lalibela were done both in an urban and rural community while our study solely conducted in a rural community might bring this great variation because mothers in urban community are near for accessing information by different media regarding timely initiation of complementary feeding compared to a rural community.

Occupation of mothers, occupation of the spouses, place of delivery and PNC visits were associated with timely initiation of complementary feeding.

Occupation of the mother is one of the factors that affected timely initiations of complementary feeding. In this case, those mothers who kept livestock were 2.21 times more likely to initiate timely complementary feeding than a housewife. This is consistent with studies conducted in Kamba and Lalibela districts (16, 23). The reason is due to Keep livestock mothers usually stay out of home with their community and they may get sufficient information about timely initiation of complementary feeding in contrast to housewife as they stay at home to wean their child early or late initiation of complementary feeding.

The occupations of the spouse were also affected timely initiation of complementary feeding. Daily labourer and merchants were more likely to initiate timely complementary feeding compared to farmers. This finding was consistent with a study done in Pakistan (24), Gode district (25). This might have happened since Merchant has better income and able to provide complementary feeding to their young infants in timely initiation of complementary feeding. Beside this merchant and daily labourer also may get a better chance of getting information regarding complementary feeding compared with the farmers.

Postnatal care user mothers’ were higher to timely initiation complementary feeding to their infants compared to those who did not follow the service. The finding is in line with Axum (26) Kamba district (16). Mothers who had no postnatal care follow up would start complementary feeding earlier before 6 months or later after 8 months compared to mothers who not followed the PNC care. A postnatal period could be an ideal time to counsel mothers on optimal complementary feeding practice Mekelle (22), Addis Ababa (20) and Lalibela District (23).

# Conclusion

The prevalence of timely introduction of complementary feeding in children aged 6-24 months is still low in study setting. The reason leads to not initiated timely complementary feeding were home delivery, unable to attend postnatal care follow-up, being housewife and farmers. This indicates that still, the community does not get information about timely initiation of complementary feeding. It needs great effort from different stakeholders to disseminate information regarding the importance of timely initiation of complementary feeding to overcome child malnutrition.

All health professionals including health extension workers should give focus to advice and counsel mothers on timely initiation of complementary feeding during antenatal, delivery and post natal period. Further research should be conducted by using qualitative study design to understand deeply socio-cultural and behavioral related factors towards timely initiation of complementary feeding.

# Declarations

Ethics approval and consent to participate**:** Ethical approval was obtained from ethical review board of (blind for peer review) with a protocol number **00310/2020**. A formal permission letter was communicated at each level. The participants were well informed about the purpose of the study and the right to refuse participation and information was collected after oral and written consent from each respondent.

Consent for Publication: Not applicable.

Availability of data and material: The data are available from the corresponding author and will be accessed as needed.

Competing interests: All authors declared that they have no competing interests and no organizations have financial or other conflicts of interest in this paper**.**

Funding: Currently**,** there are no Funding for this research, all the costs were covered by the authors.

Authors' contributions**:** MWA, ABN, ATD, TDE, CMT, NST, ABM, TKA and MYY. MWA, ABN, ATD and TKA design the study, analyzed the data, interpretation of the data, and wrote the manuscript and TDE, CMT, NST, ABM and MYY were participated in data analysis and wrote the manuscript. Finally, all the authors read and approved the manuscript. Abbreviations**:** **ANC**, Antenatal Care; **AOR**, Adjusted Odds Ratio; **CF**, Complementary Feeding; **CI**, Confidence Interval; **COR**, Crude Odds Ratio; **ETB**, Ethiopian Birr; **IYFC**, Infant and Young Child Feeding; PNC, Postnatal Care; **UNICEF**,

United Nations International Children's Emergency Fund; **WHO**, World Health Organization. Acknowledgements: We would like to acknowledge all data collectors for the success of our work.

# References

1. Giugliani ER, Cesar Gomes Victora. Complementary feeding. J Pediatr (Rio J). 2000;76(Suppl 3):S253-62.
2. Group WHO Multicentre Growth Reference Study. Complementary feeding in the who multicentre growth reference study. Acta paediatrica (Oslo, Norway: 1992) Supplement. 2006;450:27-37. 3. Cattaneo Adriano, Carol Williams, Carmen Rosa Pallás Alonso, et al. Espghan's 2008 recommendation for early introduction of complementary foods: How good is the evidence? Maternal & child nutrition. 2011;7(4):335-43.
4. Maslin Kate, Audrey Dunn Galvin, Sian Shepherd, et al. A qualitative study of mothers’ perceptions of weaning and the use of commercial infant food in the united kingdom. Maternal and Pediatric Nutrition. 2015;1(1):1000103.
5. Reilly John J, Jonathan CK Wells. Duration of exclusive breast-feeding: Introduction of complementary feeding may be necessary before 6 months of age. British Journal of Nutrition. 2005;94(6):869-72.
6. Phantumvanit Prathip, Yuka Makino, Hiroshi Ogawa, et al. Who global consultation on public health intervention against early childhood caries. Community dentistry and oral epidemiology.

2018;46(3):280-7.

7. White Jessica M, France Bégin, Richard Kumapley, Colleen Murray, Julia Krasevec.

Complementary feeding practices: Current global and regional estimates. Maternal & child nutrition. 2017;13:e12505.

8. Berhanu Mengistu, Workie Zemene, Mengistu Mekonnen. Prevalence and associated factors of nonexclusive breastfeeding to infants within the first 6 months in gondar town, northwest ethiopia, 2014.

Advances in Nursing. 2015;2015.

9. White J. M., F. Bégin. Complementary feeding practices: Current global and regional estimates. 2017 Oct;13 Suppl 2(Suppl 2). PubMed PMID: 29032623.
10. Pem Deki. Factors affecting early childhood growth and development: Golden 1000 days. Adv Practice Nurs. 2015;1(101):2573-0347.
11. Monte CM, ER Giugliani. Recommendations for the complementary feeding of the breastfed child. J Pediatr (Rio J). 2004;80(Suppl 5):S131-41.
12. "World Health Organization". Indicators for assessing infant and young child feeding practices: Part 2: Measurement. 2010.
13. Yohannes Bereket, Elias Ejamo, Thilagavathi Thangavel, Mulugeta Yohannis. Timely initiation of complementary feeding to children aged 6–23 months in rural soro district of southwest ethiopia: A cross-sectional study. BMC pediatrics. 2018;18(1):17.
14. WHO Unicef. Global strategy for infant and young child feeding. . 2003.
15. JAHANIHASHEMI	MOSTAFA	NOROOZI–ROZA	ZAVOSHY–HASSAN,	ELHAM

HADIBYGI. The timing of introduction of solid foods and food type during the first year of life in children in qazvin, iran. Journal of Food and Nutrition Research (ISSN 1336-8672). 2014;53(4):363-70.

16. Agedew E, M Demissie, D Misker, D Haftu. Early initiation of complementary feeding and associated factors among 6 months to 2 years young children. Kamba Woreda, South West Ethiopia: A

Community–Based Cross-Sectional Study J Nutr Food Sci. 2014;4(6):314.

17. Kabir Iqbal, Mansura Khanam, Kingsley E Agho, et al. Determinants of inappropriate complementary feeding practices in infant and young children in bangladesh: Secondary data analysis of demographic health survey 2007. Maternal & child nutrition. 2012;8:11-27.
18. Hibstu Desalegn Tsegaw, Dawit Jember Tesfaye, Teshome Abuka Abebo, Fanuel Belayneh

Bekele. Complementary feeding timing and its predictors among mothers’ of children aged (6-23) months old in halaba kulito town, southern ethiopia. Curr. Pediatr Res. 2018;22:61-8.

19. Andualem Atsedemariam, Afework Edmealem, Belachew Tegegne, Lehulu Tilahun, Yitayish Damtie. Timely initiation of complementary feeding and associated factors among mothers of children aged 6–24 months in dessie referral hospital, northeast ethiopia, 2019. Journal of nutrition and metabolism. 2020;2020.
20. Mohammed Shikur, Tewodros Getinet, Samrawit Solomon, Andrew D Jones. Prevalence of initiation of complementary feeding at 6 months of age and associated factors among mothers of children aged 6 to 24 months in addis ababa, ethiopia. BMC nutrition. 2018;4(1):54.
21. Semahegn Agumasie, Gezahegn Tesfaye, Alemayehu Bogale. Complementary feeding practice of mothers and associated factors in hiwot fana specialized hospital, eastern ethiopia. The Pan African Medical Journal. 2014;18.
22. Shumey Ashenafi, Meaza Demissie, Yemane Berhane. Timely initiation of complementary feeding and associated factors among children aged 6 to 12 months in northern ethiopia: An institutionbased cross-sectional study. BMC Public Health. 2013;13(1):1-7.
23. Menberu Molla 1 Tadese Ejigu, 2 and Girma Nega3,. Complementary feeding practice and associated factors among mothers having children 6–23 months of age, lasta district, amhara region, northeast ethiopia. March 2017.
24. Khokhar Saadat, Hashmat Ali Jatoi, Zohra S Lassi. Prevalence of timely introduction of complementary feeding and its related factors in children 6–24 months of age in hyderabad, pakistan.

Nursing and Midwifery Studies. 2017;6(3):115.

25. Abdi Ahmed Ali. Complementary feeding practice and associated factors among mother of children aged 6-23 months in agro-pastoral communities of rural gode district. Somali National Regional State, Eastern Ethiopia. 2018.
26. Yemane Samson, Tadesse Awoke, Measho Gebreslassie. Timely initiation of complementary feeding practice and associated factors among mothers of children aged from 6 to 24 months in axum town, north ethiopia. Int J Nutr Food Sci. 2014;3(5):438-42.

..\Documents\Supporting file.docx

Supporting information

[Click here to access/download](https://www.editorialmanager.com/pone/download.aspx?id=30040081&guid=8b546032-3774-4996-a583-e822df3ee499&scheme=1)

[Supporting Information Supporting file.docx](https://www.editorialmanager.com/pone/download.aspx?id=30040081&guid=8b546032-3774-4996-a583-e822df3ee499&scheme=1)
